# Manufacturing Technologies of Polymer Composites—A Review

**DOI:** 10.3390/polym15030712

**Published:** 2023-01-31

**Authors:** Chenchen Wu, Fan Xu, Huixiong Wang, Hong Liu, Feng Yan, Chao Ma

**Affiliations:** 1 State Key Laboratory of Robotics, Shenyang Institute of Automation, Chinese Academy of Sciences, Shenyang 110016, China; 2 Institutes for Robotics and Intelligent Manufacturing, Chinese Academy of Sciences, Shenyang 110169, China; 3 School of Mechanical Engineering and Automation, University of Science and Technology LiaoNing, Anshan 114051, China; 4 Ningbo Sunny Optoelectronic Information Co., Ltd., Yuyao, Ningbo 315400, China; 5 School of Mechanical and Engineering, Jiangsu University, Zhenjiang 210061, China

**Keywords:** polymer composites, manufacturing process, process mechanism, surface coating, additive manufacturing, magnetic pulse powder compaction

## Abstract

Polymer composites have been widely used in the aviation, aerospace, automotive, military, medical, agricultural and industrial fields due to their excellent mechanical properties, heat resistance, flame retardant, impact resistance and corrosion resistance. In general, their manufacturing process is one of the key factors affecting the life cycle of polymer composites. This article provides an overview of typical manufacturing technologies, including surface coating, additive manufacturing and magnetic pulse powder compaction, which are normally used to reduce the failure behaviour of polymer composites in service so that the quality of composite products can be improved. Advanced polymer composite powder manufacturing processes, the processing mechanism and experimental methods are described, and the influence of different manufacturing processes on the moulding quality is revealed. This investigation can provide suitable methods for the selection of manufacturing technology to improve the quality of polymer composite products.

## 1. Introduction

With the rapid development of aviation, aerospace, automobile, medical and health, military and other fields, polymer composites with their unique properties of high specific stiffness and strength, corrosion resistance, light weight and other advantages can replace traditional heavy metals, including steel and aluminium, to realise light weight and intelligence. Especially in the automotive industry, a large number of polymer composites are used to manufacture hybrid electric vehicles and electric vehicle batteries for the purpose of achieving lightweight designs [1]. Polymer composite materials are mainly constructed with reinforcement (usually high-performance fibres, such as carbon fibre, glass fibre, aramid fibre, etc.) and matrix materials (such as epoxy resin, etc.). The matrix of polymer composites is normally divided into thermoplastic and thermosetting matrices [2], both of which (see Table 1) have been widely used in aerospace, aviation, biomedicine, automotive parts, electrodes and packaging materials.

The first use of advanced polymer composites in the automotive industry can be traced back to 1953 when the first Chevrolet Corvette car was exhibited. Since then, glass fibre-reinforced plastics (GFRP) have attracted extensive attention in the automotive industry. Composite automobile grid panels, braking systems, trunk lids and body reinforcement, automobile body parts, door panels, hoods, internal structures, engine frames, T-joints, signal transmission on aircraft, aircraft wings, safety facilities and other parts have emerged, showing prospective applications of polymer composites in the lightweight design of automobiles and aircrafts [1,6,7]. Then, in the 1970s, the composites industry began to mature [8]. High-performance reinforcing materials including carbon fibres, SiC fibres, alumina fibres and aramid fibres were developed and utilised as reinforcements for resins, metals and ceramics [9] (see Table 2). The heat shield of a spacecraft [5] used phenolic resin and carbon fibre reinforced composites as fireproof and heat insulation materials. Currently, advanced composite materials are used in anything from the technology used by the defence industry to the medical field. For instance, plastics of PAEK family members are used for military and civilian purposes, among which polyetherketone ketone ketone (PEKK) composites with high mechanical properties and high temperature stability when reinforced with carbon fibres [4] are regarded as high-temperature components in aviation [10]. Polyetherimide (PEI) composites with excellent fire resistance are also used in aviation [5].

Polymer composites have gradually attracted extensive attention in biomedical applications [11] because polymers can be used as matrix and reinforcing agents in clinical applications [12]. The utilisation of polymer materials (natural and synthetic) takes into account hydrogels in scaffold fabrications. Assessment of polymer scaffold mechanical properties enables personalised patient care and prevents damage after implantation in the human body. By controlling process parameters, it is possible to obtain optimised mechanical properties of manufactured parts [13]. Their good biocompatibility, bioactivity, biostability, friction, wear performance and corrosion resistance enable them to be used in this field [14,15,16]. Thermoplastic polymers such as polylactic acid (PLA), polycaprolactone (PCL), polyglutamic acid (PGA), lactic acid/glycolic acid copolymer (PLGA) and polysanya methyl carbonate (PTMC) have become the main materials of bone scaffolds in medical surgery after compositing with nanohydroxyapatite [17]. Recently, artificial organs made of polyetheretherketone (PEEK) composite materials as implant material have been widely used in the operation of key parts of the human body [18,19], and PEKK has also been used as a new dental implant and dental restoration material [20]. After some new polymer composites have been developed, their mechanical properties, electrical conductivity and thermal conductivity have been properly controlled. It has also been widely used in electrode devices [21,22], electromagnetic interference (EMI) shielding devices [23,24,25,26], sensors [24,27,28], packaging materials [28] and energy storage devices [29].

The quality of polymer composite products is not only limited by the composite preparation but also affected by the manufacturing technology [30,31]. Based on the manufacturing characteristics of polymer composites, some typically advanced manufacturing technologies are analysed in this paper. In Section 2, the manufacturing technologies are compared, from surface to internal manufacturing, from two-dimensional to multidimensional manufacturing and from static to dynamic manufacturing. Moreover, these advanced manufacturing characteristics, mechanical behaviour and failure mechanism are discussed. In Section 3, the numerical analysis method of the polymer composite manufacturing process is discussed. Finally, remarks and prospects are presented.

## 2. Manufacturing Technology of Polymer Composites

The manufacturing technology of polymer composites is the way to obtain polymer products, embody the properties of materials and develop new materials. Traditional polymer composite moulding manufacturing technology mainly includes extrusion moulding, injection moulding, calendaring moulding, hot pressing moulding and others that are highly repeatable and capable of achieving process control and delivering high-quality components. Moulding processing has been widely used in the manufacturing of complex cross sections [32], complex structural parts [33] and sheets [34,35], which optimises the performance of composite materials to improve the life cycle of products. However, the complexity of products increases the difficulty of moulding processing. Thus, some advanced polymer composite manufacturing technologies, as shown in Table 3, such as surface coating technology [36], additive manufacturing technology [37,38,39,40] and magnetic pulse powder compaction technology [41], have been gradually developed.

### 2.1. Surface Coating Technology

Surface coating is a surface manufacturing technology that is used to coat a thin film onto the surface of a substrate material to improve the performance of the substrate. The substrate material can be a polymer material or a non-polymer material. Generally, a layer of film of other materials is coated on the surface of a polymer matrix or a layer of the polymer film is coated on a non-polymer material matrix. The former is mainly used in the biomedical field, while the latter is used as an anti-corrosion material in corrosive working environments to protect the matrix material [42]. Surface coating technology mainly includes plasma spraying [49], magnetron sputtering [50], electrochemical deposition [51] and sol–gel technology [52], as listed in Table 4.

#### 2.1.1. Plasma Spraying

**Figure 1:**
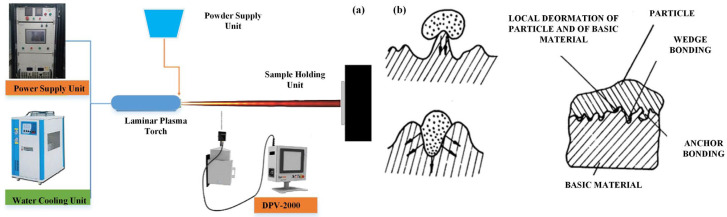
Plasma spraying technology: (**a**) schematic diagram of plasma spraying technology [58]; (**b**) deformation scheme of particles on rough substrate [60].

Table 4 shows four typical coating technologies that are currently well-developed and theoretically sound surface manufacturing technologies, among which plasma spraying has been widely applied in biomedicine [57]. It uses a plasma arc to heat the powder to a molten or semi-molten state at a temperature of tens of thousands of degrees, sprays it to the surface of the substrate at a high velocity through an injector, and forms a firm film with the substrate by mechanical combination. The film can make the surface of the substrate have the functions of wear resistance, corrosion resistance, high-temperature resistance and heat insulation, and its working principle is shown in Figure 1. First, the powder is fed into the gun by the powder-feeding gas in the feeder, and the working gas is ionised under the electric field. After gas ionisation, there are not only atoms but also positive ions and free electrons. Under the action of the thermal and kinetic effects of the plasma jet, the powder is melted and sprayed onto the substrate. Herein, the kinetic energy is converted to thermal and deformation energy during the impact of flowing molten particles on the substrate and solidifies into films [58].

The film and substrate mainly exhibit mechanical interlocking combined with physical and chemical adsorption [59,60]. The bond strength between the film and the substrate is affected by the roughness of the substrate surface, and publications have shown that the bond strength is proportional to the roughness of the substrate surface [61]. In the process of particle droplets contacting the rough substrate surface, the particle droplets wedge into the gap of the substrate surface and firmly combine with the substrate surface, as shown in Figure 1. Figure 1a shows a schematic diagram of plasma spraying technology, which includes a power supply unit, water cooling unit, sample holding unit, powder supply unit and plasma torch, and the spraying parameters of the laminar plasma spray system are shown in reference [58]. At the same time, the contact between the particle droplets and the convex peaks on the substrate surface leads to slower heat dissipation and prolonged constant temperature time, which is enough to form chemical bonds between the particles and the substrate surface [60], as shown in Figure 1b. Therefore, roughening the substrate surface can obtain higher bonding strength.

Factors influencing the coating performance of plasma spraying technology have been described in multiple papers. Rubarsky et al. [60] conducted experimental studies on the physical properties of plasma coatings, such as porosity, bonding strength, coating thickness, hardness, electrical conductivity and thermal conductivity, which proved that the spraying velocity has a great influence on the porosity of the coating, and the higher the spraying velocity is, the smaller the porosity. At the same time, the properties of the coating materials have a great influence on the porosity and bonding strength. The porosity of the coating of brittle materials is larger than that of plastic materials, but the bonding strength is smaller than that of plastic materials. The thickness of the coating has the greatest influence on its performance. With the increase in the thickness of the film, the thermal conductivity decreases, the internal stress increases, and cracks occur. Simultaneously, the strength, hardness and conductivity of the film are reduced, so the coating should be as thin as possible without sacrificing performance. In addition, the size of powder particles should be limited within a certain range [49]. Uneven particle size affects the uniformity of heating, leading to the existence of unmelted powder particles, which seriously affects the coating properties [62].

#### 2.1.2. Magnetron Sputtering

**Figure 2:**
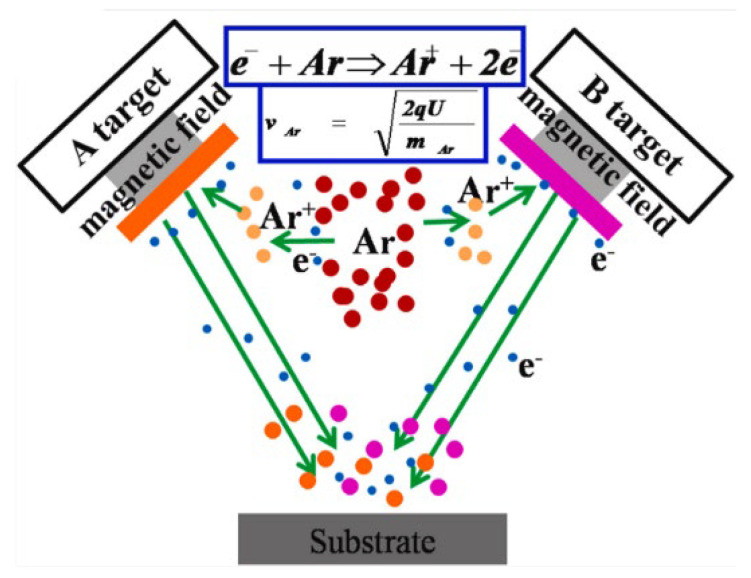
Magnetron sputtering [63].

Different from plasma spraying, magnetron sputtering is the collision process between the incident particle and the target, and its working principle is shown in Figure 2 [63]. The incident particle undergoes a complex scattering process in the target, colliding with the target atom and transferring some of its momenta to the target atom, which in turn collides with other target atoms, forming a cascade process. Some target atoms near the surface obtain enough momentum to move outwards and leave the target to be sputtered onto the substrate surface to complete the deposition process. However, the growth process of thin films and the bonding form between thin films and substrates are very complicated, so far, there is no unified theory to explain the physical mechanism related to the growth process [64].

The roughness of the coating has a great influence on the service performance of the product [65,66], and the uneven surface of the coating will scratch the parts and make them scrapped in advance. There is always internal stress in the coating during the deposition process, which easily causes cracks. Meanwhile, there is stress concentration in the sharp corners of the coating, which makes it easy to crack. Nevertheless, the cracks can be reduced by coating multilayer plastic and tough materials or annealing treatment after coating. Annealing can not only reduce the roughness and internal stress but also improve the conductivity and bending strength of the coating.

In comparison to physical vapour deposition methods, such as evaporation, molecular beam epitaxy (MBE) and pulsed laser deposition (PLD), although magnetron sputtering yields a wider range of target selection, faster deposition rate, lower equipment cost and less damage to the substrate [61], the deposition rate is relatively low, which limits its application in industry. Moreover, the uniformity of the coating is slightly worse than that of MBE and PLD, and it can reach the nanometre level efficiently [67]. Researchers often study deposition parameters such as the shape of equipment, particle mass, particle ionisation, particle charge and particle composition to obtain the best deposition rate [64]. The high-power pulsed magnetron sputtering technology developed in recent years combines the advantages of high-energy deposition with magnetron sputtering to realise faster film deposition. Meanwhile, compared with DC magnetron sputtering, the deposited films have higher density [68], better optical properties [69] and stronger adhesion and toughness [70], yielding potential applications in the biomedical field.

#### 2.1.3. Electrophoretic Deposition or Electrochemical Deposition

**Figure 3:**
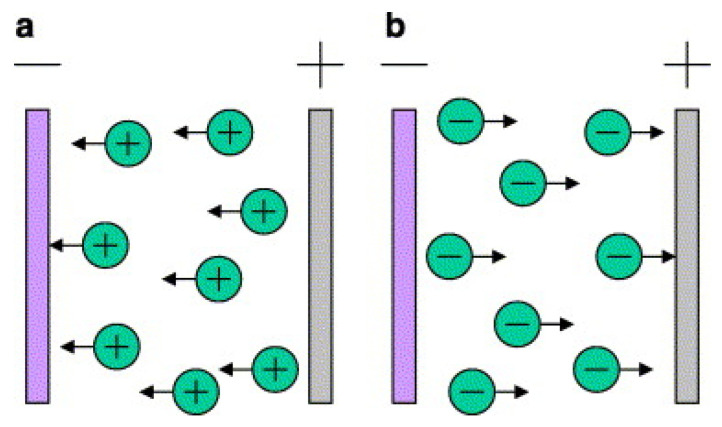
Electrophoretic deposition. (**a**) Cathodic EPD and (**b**) anodic EPD [74].

Aiming at the problem that the working temperature of plasma spraying and magnetron sputtering is too high, electrophoretic deposition technology is favoured due to its low- and medium-temperature working conditions [30]. Electrophoretic deposition is a colloid treatment technology that includes electrophoresis and deposition processes [71], and its working principle is shown in Figure 3. Electrophoresis refers to the movement of the charged particles in the suspension towards the relatively charged substrate under the influence of the applied electric field, while in the deposition process, the particles condense on the surface of the conductive substrate to produce a uniform coating. In the past few decades, this technology has been widely used in the biomedical field [36,72]. Electrophoretic deposition technology can be used to deposit any granular material, but there is no unified theory to explain its deposition mechanism [73]. According to the experimental results, researchers inferred different deposition mechanisms [74,75], but determining the theoretical model is still an unresolved problem at present.

**Figure 4:**
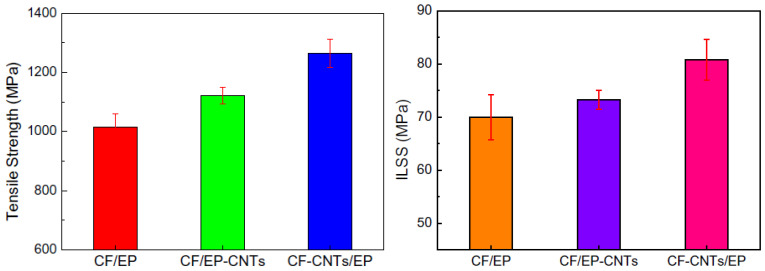
Tensile and interlaminar shear strength of CF/EP, CF/EP-CNTs and CF-CNTs/EP [77].

In addition, electrophoretic deposition is more flexible than plasma spraying and magnetron sputtering, which can control the thickness of the film at will by controlling the deposition time, voltage and concentration of particles in the solution [74,76]. The working temperature of electrophoretic deposition is much lower than that of plasma spraying and magnetron sputtering; thus, it will avoid the generation of thermal stress in the deposition process and depositing films on substrates with complex shapes [74]. Zhao et al. [77] dealt with the carbon nanotube/epoxy resin composite with an epoxy resin film deposited on the carbon nanotube substrate and found that the shear and tensile strength of the carbon nanotube/epoxy resin composite were significantly improved in comparison to the carbon nanotube material, as shown in Figure 4. Functionalised CNTs, treated by either chemical or physical treatments, can promote CNT dispersion and stress transfer between CNTs and matrices. Compared with CF/EP, increases of 10.41% (tensile strength) AND 15.47% (IFSS) were observed for CF/EP-CNTs, and improvements of 24.42% and 4.77% (ILSS) were observed for CF-CNTs/EP [77]. They showed that carbon nanotube/epoxy resin composites have better mechanical properties than carbon.

However, the shear and tensile strengths of carbon nanotube/epoxy resin composites modified by the matrix are much larger than those modified by the interface, which limits the application range of electrophoretically deposited films. In addition, the electrophoretic deposition composite coating had better performance than the single material coating. For instance, regarding a protective layer of copper, RGO-PVC monomer composite coatings deposited by electrophoresis had higher corrosion resistance and adhesion strength than pure PVC monomer coatings [78]. The nanohydroxyapatite/polylactic acid–glycolic acid copolymer film deposited by electrophoresis also exhibited higher corrosion resistance and adhesive strength than the nanohydroxyapatite film, which shows that the performance of the composite coating is better than that of a single pure coating [79].

#### 2.1.4. Sol–Gel Technology

**Figure 5:**
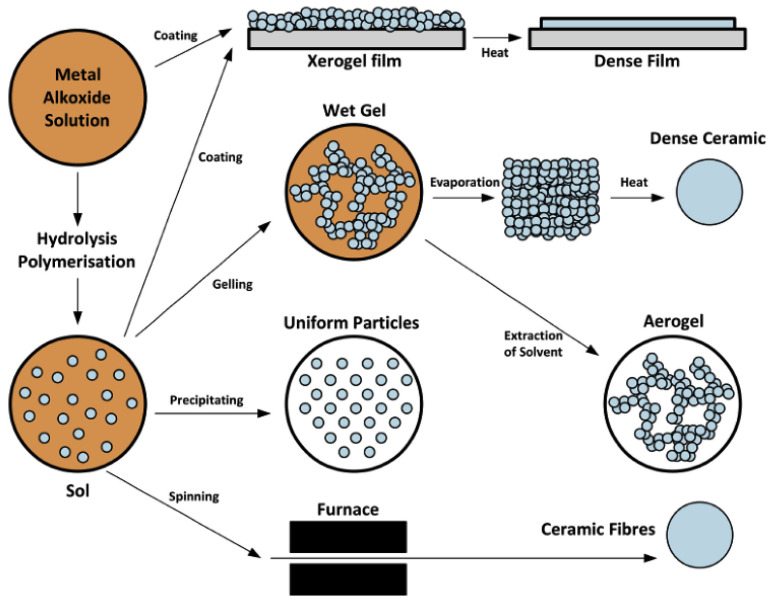
Schematic diagram of sol–gel technology [36].

Sol–gel technology, first reported by Yamane et al. [80], has been rapidly developed and used to prepare oxide thin films [81], and its working principle is shown in Figure 5. As already known, the first step for deposition of the coating with any method is pretreating (cleaning) the substrate because the surface of the substrate has a great influence on the structure and physical properties of the film [82]. The types and properties of the substrate will also directly affect the structure and performance of the film. Most polymer resins have low surface free energy and lack polar functional groups, which leads to poor adhesion [83]. Therefore, it is necessary to carry out surface pretreatment on the substrate to improve the adhesion between the film and the polymer substrate, such as ionic functionalisation [84] and chemical abrasion [85]. Yamaguchi et al. [86] prepared alumina films on polyethylene terephthalate (PET), polycarbonate (PC) and polymethyl methacrylate (PMMA) substrates with light reflectance of 1.5%, 1.0% and 0.8%, respectively, indicating that substrates of different materials have a great influence on the properties of the prepared films.

The second step is film coating. The sol–gel method can be divided into two groups: sol–gel dip-coating and sol–gel spin-coating [82], and the wet gel can only be dried and sintered before the final film can be obtained. Due to the large amount of liquid in the wet gel, it shrinks and cracks easily during drying. Under normal circumstances, controlling the ambient atmosphere and maintaining a certain relative humidity during drying treatment can obviously improve film cracking [87]. The thin film and the substrate are bonded by chemical bonds [88]. This bonding mode only occurs in atoms or groups close to the surface film of the substrate, while polymerisation or contraction occurs between particles on the surface of the principal substrate, resulting in a large shrinkage rate and easy cracking. Therefore, the thickness of the film should be carefully controlled.

Due to the high temperature of the plasma spraying and magnetron sputtering film preparation process, the film prone to oxidation is generally coated under vacuum conditions, while in contrast, sol–gel technology does not need to be coated under a vacuum, and the working temperature is lower. However, sol–gel technology is prone to cracking of the film during the drying process, and the preparation time is too long [82]. Although the basic principles of the above technologies are similar, there are still some differences. Different coating technologies can be adopted for different materials, equipment and application fields.

### 2.2. Additive Manufacturing

Additive manufacturing, also known as rapid manufacturing technology, is a multidimensional manufacturing technology that stacks materials layer by layer, which generally refers to 3D printing technology [89]. On the basis of 3D printing technology, 4D and 5D printing have been developed, in which 3D printing technology is already quite mature, but 4D and 5D printing technology only used in some high-end customised products are less mature at present [90].

#### 2.2.1. Three-Dimensional Printing

**Figure 6:**
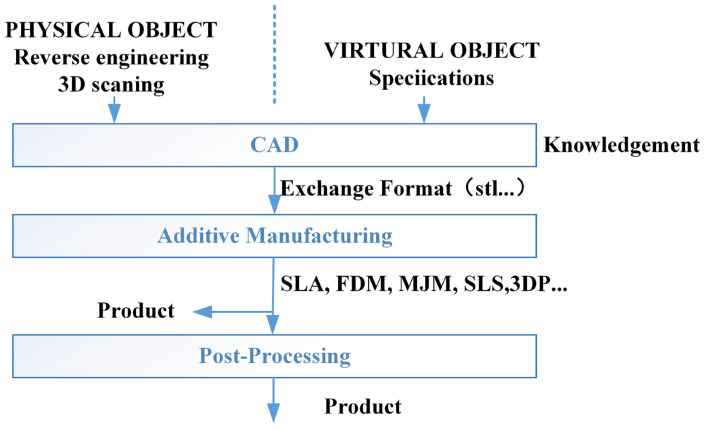
Three-dimensional printing process [91].

**Figure 7:**
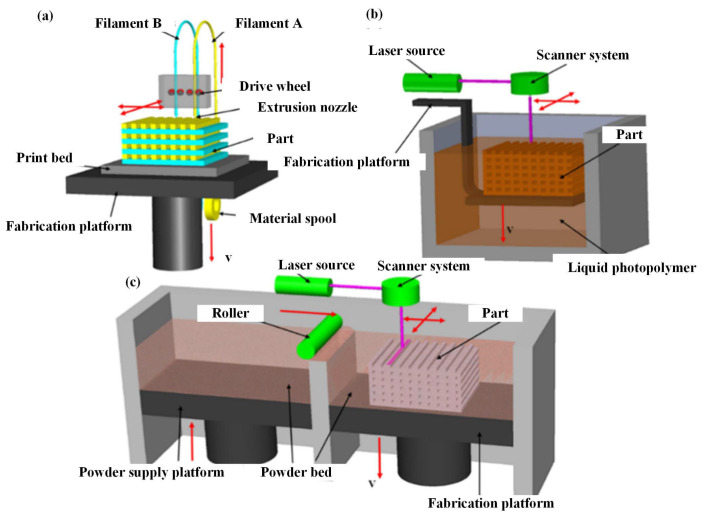
Schematic diagram of typical 3D printing [93]: (**a**) FDM; (**b**) SLA; (**c**) SLS.

Three-dimensional printing is a prototyping technology using powdery metal, plastic and adhesive materials to print objects layer by layer (see Figure 6 [91]), and its working principle is shown in Figure 7. Different from the surface coating, 3D printing is not an independent technology but integrates computer, computer-aided design, laser, printing and numerical control processing technologies [92]. Three-dimensional printing has developed rapidly with the development of these technologies, and different forms of 3D printing methods (see Table 5), including fused deposition manufacturing (FDM) [93], selective laser sintering (SLS) [94], powder bed and inkjet head 3D printing (3DP) [95] and stereolithography (SLA) [96], have gradually emerged. FDM adopts a hot extrusion process to extrude the filamentous thermoplastic-based polymer composite material into a molten state and deposit and solidify. SLS and 3DP are processed with powdered raw materials, the powders are bonded together in one layer, and then the next layer of powders is laid and bonded until printing is completed. SLS uses laser scanning and thermally induced powder sintering to bond the powders through molecular diffusion [97], while 3DP bonds the powder by adhesive. Different from other powder processing methods, the raw material of SLA is mainly liquid photocurable polymer. The UV laser moves on the controlled path, the irradiated photocurable polymer is polymerised and cured, and then the moving platform is lowered to cure the next layer of the photocurable polymer [98]. Furthermore, unlike the traditional subtractive manufacturing process, 3DP technology can avoid waste and achieve a near-net and accurate shape [99], but the work efficiency is low, and the printing of the curved layer is still difficult. Three-dimensional printing technology can print some thermoplastic polymer materials [100,101,102], but most pure polymer materials have difficulty achieving ideal mechanical performance [97,103].

Some new special materials for 3D printing can be prepared to improve the quality of polymer composite parts. Laboratory research findings have demonstrated that by adding synthetic particles to increase the stiffness of polymer composites [104], adding Al_2_O_3_ to improve wear resistance [105], or adding ceramic particles to improve the dielectric constant [106], the performance of products has been further improved. For instance, Mohammadizadeh et al. [107] used nylon as the matrix and carbon fibre and glass fibre as reinforcing agents to obtain parts through 3D printing, yielding even better performance than metals. Fibre-reinforced polymer composite parts with complex structures are easier to produce by 3DP (3D printing) technology than other fabrication methods. Nanomaterials are also often used as additives in polymer matrices to enhance the mechanical properties of composites. An available publication [108] proved that the addition of 5 wt% nano-TiO_2_ contributed to a 13.2% increase in its tensile strength, but its elongation and toughness were significantly decreased while its brittleness increased. Therefore, it is important to select suitable additives for obtaining high-quality products, which can reduce or avoid failure behaviour during manufacturing and service.

Although reinforced materials can significantly improve the performance of parts, reinforced materials often increase material anisotropy and porosity [109], and the anisotropy of parts makes the design more complicated [110]. FDM is the most widely used 3D printing technology at present, and it has been found that nozzle size and printing speed significantly affect the anisotropy of parts [110]. After the addition of reinforcement, the porosity of the part is significantly increased due to the poor interface of the reinforcement and the polymer [109,111,112], which will lead to stress concentration. Thus, the research focus of 3D printing is to adjust the processing parameters to achieve lower porosity. Klift et al. [113] found that the porosity increased as the number of carbon fibre layers increased, while the tensile strength of carbon fibre-reinforced polymers decreased. Recently, researchers have found that adding expandable microspheres to polymers can reduce the porosity of printed parts [97]. Some publications have shown that in situ consolidation techniques can also be used to reduce porosity [114]. Additionally, the action of coupling fields, such as external magnetic fields [115] and vibration fields [116], can achieve better control of porosity by adjusting the arrangement of reinforcement in the matrix material, but these techniques are not applicable to all materials. In addition to avoiding the anisotropy and stress concentration defects produced by pores, Duigou et al. [117] used the water absorption of the pores of FDM printed parts to produce the original self-bending device driven under a humidity gradient.

#### 2.2.2. Four-Dimensional Printing

**Figure 8:**
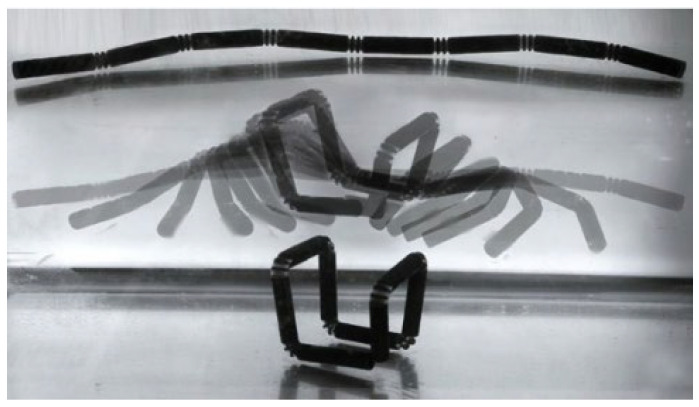
Self-folding line [90].

Four-dimensional printing technology with increased time dimensions has been developed in the past decade since it was found that the shape of three-dimensional printed products changes autonomously over time, as shown in Figure 8 [118], which can overcome the shortcomings of slow three-dimensional printing. Four-dimensional printing helps products realise shape changes with time under external stimuli such as light, electricity and temperature. Zhang et al. [119] printed a shape memory polymer composite triggered by a magnetic field and found that the printed structure can be quickly restored to its original shape, and the temperature during the entire restoration process is approximately 40 °C, which is in line with the temperature of the human body. Zeng et al. [120] conducted electric heating 4D printing on continuous carbon fibre-reinforced shape memory polymer composites and found that the shape recovery rate was as high as 95%. Four-dimensional printed products are normally conceptual models, but they are still attractive due to their self-assembly, self-adaptation and self-repairing characteristics. Additionally, printed shape memory materials will promote the development of biomedicine [121,122]. However, 4D printing is relatively harsh and requires smart materials, which greatly limits its application. Additionally, for the printing of complex curved layers, it is still an obstacle that 4D printing technology cannot overcome.

#### 2.2.3. Five-Dimensional Printing

**Figure 9:**
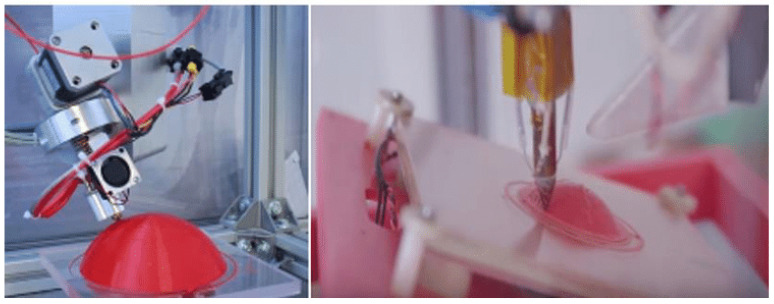
Five-dimensional printed pressure cap [123].

Five-dimensional printing can print products through five axes, as shown in Figure 9, to realise printing at any angle. The curved layer can be easily printed, which solves the problem that complicated curved layers cannot be obtained by 3D and 4D printing. Additionally, the products printed by 5D printing possess high strength. Five-dimensional technology qualifies printing more complex structures [90,123], and it has undoubtedly become a good choice for the manufacturing of complex structural parts with requirements for high strength [124].

The difference between 3D, 4D and 5D printing technology is that 3D printing can only be moved in the plane to achieve layer-by-layer printing, while 5D printing can achieve printing at any angle. Four-dimensional printing is a special method that does not achieve four-axis printing like five-dimensional printing but increases the dimension of time, and the materials to achieve four-dimensional printing must be smart. Recently, 4D and 5D printing technologies have been combined to propose 6D printing technology [125] by adding a time dimension on the basis of 5D printing technology, which can not only realise printing at any angle but also overcome the disadvantages of slow 3D printing. Perhaps this manufacturing technology will successfully mould parts with complex shapes in the future.

### 2.3. Magnetic Pulse Powder Compaction Technology

**Figure 10:**
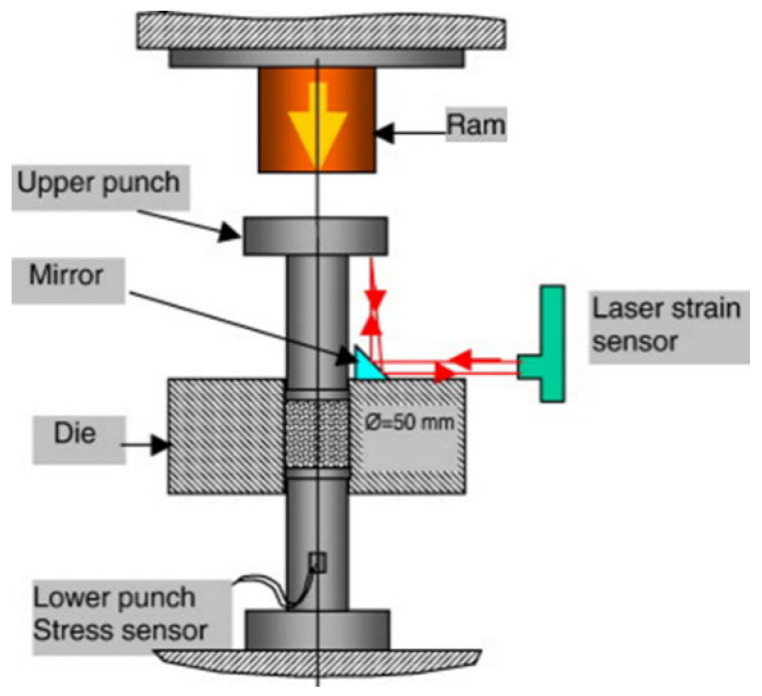
Schematic diagram of high-velocity compaction [129].

The key indicator to measure the quality of powder moulding is compaction density. The extrusion moulding, injection moulding, calendaring and hot press moulding manufacturing technologies mentioned above are all quasistatic moulding, and an uneven density distribution of compacts is prone to occur during the moulding process. The friction between the powder particles and the die leads to energy loss, which causes the green compact to generate large residual stress during the sintering process and induce cracking of the part. Magnetic pulse powder compaction technology is a rapid prototyping method that belongs to a dynamic high-velocity compaction process. Similar to explosive compaction and other high-velocity compaction technologies, the powders are compacted through stress waves, as shown in Figure 10. Previous publications have recorded the applications of high-velocity compaction technology for polymer composites [126,127,128]. Compaction velocity is a key factor influencing product quality, and how to control explosive compaction velocity has always been a technical problem. Controlling the release of the heavy hammer height can achieve the purpose of controlling the compaction velocity, but noise pollution is unavoidable [129].

**Figure 11:**
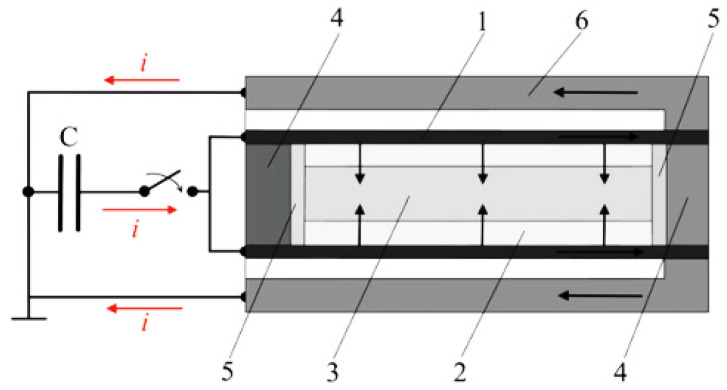
Schematic diagram of magnetic pulse powder compaction: principle scheme of magnetic pulse compaction of tubes: 1 copper shell, 2 powder, 3 steel rod, 4 plug, 5 spacer, 6 reverse conductor [130].

Magnetic pulse powder compaction technology is a high-velocity compaction technology in which a strong pulse magnetic field is generated by the instantaneous discharge of a discharge capacitor to the coil, thereby forming a magnetic field force and pushing the punch to compact the powder. Magnetic pulse powder compaction technology can be divided into radial compaction [130] and axial compaction [131], and its principle is shown in Figure 11. Magnetic pulse powder compaction technology under the action of stress waves can mould polymer composite powders, and the compaction velocity can be controlled by controlling the discharge time and/or discharge voltage to avoid noise pollution caused by heavy hammer impact.

In Figure 11, the powder is packed into a conductive container, the reverse conductor passes a high-frequency pulse current to form a high-frequency magnetic field, and the copper shell generates an induced current, which interacts with the high-frequency magnetic field to generate radial pressure to compact the powder. In reference [131], the discharge capacitor discharges to the inductor (coil), and the working coil flows through the impulse current to form a high-frequency magnetic field around the working coil. The metal plate generates an induced current, which interacts with the high-frequency magnetic field to generate axial pressure, and the metal plate drives the punch to move downwards to compact the powder. During the powder compaction process, the powder particles successively passed through the rearrangement stage, the elastoplastic deformation stage and the fracture stage. The large deformation generates a large amount of heat on the surface of the particles and forms local welding. The magnetic pulse compaction process is extremely fast in a few milliseconds or even a few microseconds, and the air between the particle gap is too late to be eliminated in the particle gap to form high-temperature and high-pressure heat-insulating gas, which generates a large amount of heat when passing through the particles, causing local welding between the particles.

During the magnetic pulse high-velocity compaction process, as the compaction rate increases, the friction coefficient between the powder particles and the die will decrease, thereby reducing the reduction in the compaction force caused by friction loss and ultimately making the density distribution more uniform [132]. At the same time, the die wall lubrication will make the compaction density higher and more uniform [133]. Therefore, the selection of an appropriate lubricant is a prerequisite for obtaining high-density billets.

Research on magnetic pulse powder compaction technology is mainly aimed at metal or metal composite materials [134,135,136,137,138], inorganic non-metallic materials [139,140,141] and other powder materials with good electrical and thermal conductivity and mainly studies the influence of process parameters on the quality of the compact, such as the discharge energy [57], impact speed and load [142], friction coefficient between powder and die [133], compaction temperature [135], number of compactions [143], compaction method [131] and initial relative density [144]. There are very few discussions on polymers or polymer composites. Wang et al. [41] studied the powder moulding process of graphene/PEKK composites using magnetic pulse powder compaction and obtained the influence rule of the main process parameters on the quality of graphene composites by magnetic pulse compaction but lacked a theoretical model.

Many factors affect the quality of magnetic pulse compaction products. In addition to the process parameters, the basic properties of raw materials are the key influencing factors, such as the electrical conductivity, thermal conductivity and mechanical properties of the material. To predict the distribution of the stress field, strain field and damage field of materials in the process of manufacturing and service and to reduce product cost, finite element numerical simulation analysis is an indispensable method. Wang et al. [41] carried out a simulation of the moulding process, preliminarily determined the process parameters that affect the quality, and revealed the law of impact force on the quality of the green compact. The relationship between the impact velocity and impact load on the quality of the compact and the conclusion that the density is proportional to the discharge energy are obtained, which provides a theoretical basis for experimental research.

Magnetic pulse powder compaction has the advantages of high density and uniform distribution, strong comprehensive performance, low elastic aftereffect, low cost and high productivity. However, the energy utilisation rate of equipment is extremely low, and most of the energy is consumed by the magnetic field penetrating into the workpiece, the coil resistance heating and the residual energy of the magnetic field is converted into heat. Although the energy loss can be reduced by reducing the penetration depth of the magnetic field and winding the coil with low resistance, the energy utilisation rate still cannot achieve the expected effect, so energy recovery and utilisation are still difficult.

## 3. Numerical Simulation of the Polymer Composite Manufacturing Process

During the manufacturing process of polymer composites, online real-time monitoring cannot be carried out due to the characteristics of equipment, working environment and product shape, etc., and failure behaviour and evolution process cannot be predicted in time. Therefore, the establishment of a finite element numerical simulation model can effectively and accurately describe the product manufacturing process at a relatively low cost. Some studies have analysed defects from the manufacturing process of polymer composites by surface coating, additive manufacturing and magnetic pulse powder compaction technology to predict the evolution of defects during the manufacturing process [145,146]. In this paper, the manufacturing process of polymer composites is sorted out, as shown in Table 6, summarising the technical methods used by previous researchers at the polymer composite manufacturing research institute.

### 3.1. Surface Coating Technology

The main defects of surface coating technology are pores and cracks in the coating. Numerical analysis can be used to understand the failure mechanism and improve the quality of coating products, and it is crucial to develop a numerical model that can describe the evolution of microstructure defects during the manufacturing process. The most common failure of surface coatings is cracking and shedding due to residual stress, which affects the integrity, performance, service life, fatigue performance and corrosion resistance of coating parts [165]. Although the residual stress can be measured by X-ray diffraction (XRD) [166], neutron diffraction [167], curvature measurement [168], open-loop test [169] and nanoindentation [170], the finite element numerical simulation method is simple and effective to reduce the cost, and it can analyse the failure evolution mechanism under the coupled field. Ferguen et al. [147] established a discrete element (DEM) numerical model to simulate the crack propagation of a plasma sprayed Al_2_O_3_ coating by the C++ program, and the results showed that cracks initiated inside the coating or in the interface between the coating and the substrate. If the crack initiates inside the coating, the crack will extend vertically to the coating surface. If crack initiation occurs at the interface between the coating and the substrate, the crack will evolve under the interaction of other cracks, which will lead to the coating peeling off. When two kinds of cracks work together, the failure of the coating is more obvious. Lin et al. [148] obtained the influence law of interface bonding on residual stress by single particle and multiparticle collision simulation and found that the balance between compressive stress caused by shot peening and relaxation caused by the bonding process was the main reason for residual stress.

To prolong the service life of the coating, it is necessary to reduce the residual stress of the coating and avoid cracking and premature failure. A large number of researchers have carried out studies on the influence of process parameters and particle properties on residual stress. Shayegan et al. [149] established single-particle and multi-particle collision models using LS-DYNA and verified the correctness of the models by an X-ray diffraction test on the substrate surface. The effects of the impact velocity, particle diameter, impact angle, particle shape and friction between the particle and the substrate on the residual stress were studied, and finally, the optimal parameters were determined. Yusof et al. [150] simulated the effect of spherical and elliptic particles on the substrate by smooth particle hydrodynamics (SPH) and the Johnson–Cook model and found that the tensile stress in the coating deposited by elliptic particles would damage the quality of the coating; however, the compressive stress inside the coating deposited by spherical particles will increase the fatigue life of the coating. Zhuang et al. [151] analysed the influence of the MgO coating thickness and convective heat transfer coefficient on the residual stress using the thermomechanical coupling module of ANSYS.

Stress analysis of the numerical simulation demonstrated that reducing the coating thickness had a positive effect on the reduction of residual stress, and the higher convective heat transfer coefficient and shorter cooling time were more favourable for the coating. Meng et al. [152] used the finite element method to simulate the scratching behaviour of copper films prepared by modulated pulse power magnetron sputtering technology, analysed the influence of sputtering pressure on the scratch behaviour, and found that the delamination of copper films became more serious with increasing sputtering pressure. Nemeth [153] simulated and analysed the influence rule of filler particle size on the performance of aluminium coatings containing silica filler prepared by the sol–gel method using the commercial finite element software Elastica. It was found that the increase in particle size contributed to the increase in the critical thickness of the coating, and there was no crack in the critical thickness range. In this case, the greater the thickness of the coating was, the stronger the scratch resistance of the coating. In addition, the surface morphology of the substrate, the temperature of the substrate and the temperature of the particles had a greater influence on the residual stress of the coating. Xie et al. [154] simulated the interface morphology of the SiC transition layer by ANSYS APDL, considering the influence of shape, roughness and curvature on the residual stress of a plasma-sprayed ZrC-based coating. A stress gradient at the sine wave interface with only one valley was found to be the smallest, which was not prone to cracking. Additionally, the residual stress was proportional to the interface curvature, which was a smooth plane, and cracks were most likely to occur when the interface curvature tended to infinity. Song et al. [156] carried out a single particle collision simulation using the Lagrangian finite element method (FE) in ABAQUS/explicit and analysed the influence of particle and substrate temperatures on the adhesive strength of the coating. It was found that the higher the particle temperature was, the higher the adhesive strength, and there was an optimal substrate temperature that adversely affected the adhesive strength if it exceeded this temperature.

### 3.2. Additive Manufacturing

The main defect of additive manufacturing is the porosity and residual stress in the billet. Due to the poor interface combination between the reinforcement material and the polymer, the porosity of printed parts increases significantly after the addition of the reinforcement material, which leads to stress concentration and premature failure of the parts. Porosity is an important factor affecting the service quality of products, and it can rarely be reduced by postprocessing. The main method of porosity measurement at present is image collection, but the measurement of porosity in the unit and simulation of product pores within a product are still difficult. At present, there are few reports about the simulation of product porosity. Moumen et al. [112] proposed a numerical method called the representative volume element (RVE), which is a cubic element in which single or multiple particles are randomly or uniformly embedded in a polymer matrix with a certain volume fraction similar to that of printed composite materials, and proposed two algorithms of random sequential adsorption (RSA) [171] and the Poisson process [172] to define particles in space.

Another important factor affecting the failure behaviour of parts is the residual stress, which is caused by the repeated deposition of hot materials into cold materials [156]. Although the residual stress is small, it is magnified on a large scale, and even a small thermal strain may cause deformation of several millimetres or even larger. Compton et al. [156] established a one-dimensional thermal finite difference model to simulate the manufacturing process of large-scale thermoplastic-reinforced thin-walled profiles. To prevent delamination and cracking, the top temperature should be kept above the glass transition temperature (Tg). Charoula et al. [157] established a real-time monitoring system, which was essential for the evaluation and control of the final part quality; at the same time, the accuracy of the system was experimentally and numerically verified. Utilising the monitoring system, the parameters of the moulding manufacturing process could be adjusted in real-time, the thermal gradient and residual stress could be effectively controlled, and problems such as part deformation, inaccurate size and even moulding manufacturing failure could be avoided. Ghorbani et al. [158] used the finite element software ANSYS to establish a thermodynamic model to simulate the part deformation and residual stress distribution during the deposition process, and large thermal gradients and stress accumulation traces at the corners of the tool path were found. Ji et al. [159] established a three-dimensional transient thermal nonlinear finite element model using the ANSYS parametric design language APDL to simulate the temperature distribution and found that the temperature gradient near the edge of the manufactured part was the largest, which led to the largest residual stress gradient and easy cracking of the part. Residual stress is released under the stimulation of the external environment, which affects the structure and material deformation of 4D printed products. Yu et al. [160] proposed sequential residual stress estimation and sequential deformation simulation methods to simulate the deformation of designed products and verified the accuracy of the model through a large number of experiments. To speed up the computation, a graphical neural network (GNN) was considered, and a simulation method with mechanical accuracy and calculation efficiency was explored based on machine learning to make appropriate abstractions and simplifications. The residual stress can be effectively reduced by adjusting the process parameters. Zhou et al. [161] simulated the FDM deposition process with ANSYS to predict the distribution law of thermal stress and temperature in the process. It was found that reducing the extrusion temperature and the layer thickness and slowing down the printing speed can reduce the vertical deformation and residual thermal stress, and the accuracy of the model was verified by quantifying the temperature in the deposition process with infrared sensors. Although an increasing number of researchers have focused on the numerical simulation method of the additive manufacturing process, the complexity of the current printing process also brings many difficulties to numerical simulation [173]. In 2019, Siemens launched an additive manufacturing process simulation tool PLM for predicting the stress concentration and deformation of samples during the printing process. The software uses a digital twin to simulate the printing process to predict the deformation during the printing process and automatically generate the corrected geometry to compensate for deformation. It not only helps to alleviate the part deformation, structural problems and printing stops caused by residual stress but also achieves the efficiency required for a fully industrialised additive manufacturing process [162].

### 3.3. Magnetic Pulse Powder Compaction Technology

The biggest problem of quasistatic powder compaction technology lies in the uneven density distribution, while magnetic pulse compaction technology can significantly improve the powder compacting density distribution, but as a result of magnetic pulse powder compaction being passed in the form of stress waves, pressure loss is inevitable, especially for large, compacted parts, which leads to the uneven density distribution of green compacts and easy cracking. Density is the most important index that affects the performance and life of parts compacted by magnetic pulse powder compaction technology. From static compaction to dynamic compaction and from cold compaction to warm compaction, the density and density uniformity of the compacted parts are improved. Only a few numerical simulations are available on magnetic pulse powder compaction technology. Li et al. [163] realised joint simulation with ANSYS/Multiphysical and ABAQUS/Explicit and established an improved Drucker–Prager Cap model using the VUSDFLD subroutine. The velocity, pressure change, final relative density distribution and relative density uniformity of the magnetic pulse radial compaction of W-Cu_20_ powder were predicted by using this model, and the correctness of the model was verified through experiments. Although the slit of the shape after compaction caused an uneven density distribution, the degree of unevenness was tiny. Olevsky et al. [164] established a theoretical model of the pulsed magnetic field and mechanical system dynamics, considered the phenomenological constitutive model of a powder hardening plastic body to simulate the process of uniaxial magnetic pulse compaction of nanopowder and studied the influence of processing parameters such as the quality of the dense powder, the quality of parts of accelerating compaction equipment and the diameter of the sample on the compaction density. By adjusting the process parameters, the best combination of parameters and compact parts can be obtained.

## 4. Summary and Prospect

Polymer composites have a wide range of applications in aerospace, aviation, biomedicine, automatic parts, electrodes, packaging materials and other fields. Traditional manufacturing processes such as injection moulding, rolling moulding, extrusion moulding and hot pressing cannot meet these needs. This paper reviews the advantages and disadvantages of surface coating technology, additive manufacturing technology and magnetic pulse powder pressing technology, as well as the factors affecting the performance of polymer composites (see Table 7).

However, these manufacturing processes are still facing some challenges, and the defects existing during the manufacturing process have become a problem that needs to be considered. The residual stress in the surface coating process, the pores and thermal stress caused by additive manufacturing and the uneven density distribution of the compact under the magnetic pulse powder compaction process seriously affect the failure behaviour of the product. Finite element simulation can effectively describe the product manufacturing process to judge the failure behaviour and predict the failure evolution process to reduce costs. Although research on defect analysis in the manufacturing process is still insufficient, FEA is undoubtedly an effective method for analysing the structure of composite materials and predicting the failure behaviour, especially for developing simulation tools.

In the future, intelligent manufacturing will assist these advanced manufacturing techniques in processing polymer composites. Due to the rapid development of intelligent manufacturing, some robots have been used to manufacture polymer composite parts in terms of labour cost control and reduction of production defects. The combination of traditional manufacturing technology and artificial intelligence will be prospective, which can not only detect defects in real time but also compensate for defects.

## Figures and Tables

**Table 1:** Properties of polymer composites.

Polymers	Types	Properties	Applications
Epoxy [3]	Thermosetting matrix	Excellent physical, mechanical and electrical insulation properties, bonding properties, processing flexibility, brittle	Coatings, composite materials, casting materials, adhesives, moulding materials and injection moulding materials, electronics, civil engineering, aviation, automotive
Polyester resin [1]	Thermosetting matrix	Good manufacturability, curing at room temperature, moulding at normal pressure	General civil industry, automotive, ship, chemicals, electronics
Phenolic resin [3]	Thermosetting matrix	Cheaper than epoxy, good resistance to high temperature, water and acid, high shrinkage rate	Powder pressing plastics, glass fibre reinforced plastics, ablative materials, interior decoration, electrical engineering materials
PAEK [4]	Thermoplastic matrix	Excellent properties in mechanics, high-temperature, corrosion and UV resistance.	Aviation, automotive, electronic and mechanical components subjected to a harsh environment
PEI [5]	Thermoplastic matrix	Heat resistance, inherent flame retardancy, extremely high strength and stiffness, corrosion resistance, excellent performance in moulding processing	Tableware, medical apparatus and equipment, aviation, automotive

**Table 2:** International representative schemes of materials [9].

Country	Time Period/Year	Schemes	Critical Focus
US	1990–1999	The Advanced Technology Program	Composite materials; material processing technology in heavy manufacturing industry
EU	2007–2013	Seventh Framework Programme	Cutting-edge technologies of advanced materials; nanotechnologies, materials and production
Japan	2010	Japan’s Industrial Structure Outlook 2010	Nanomaterial; carbon fibres; functional chemicals
US	2013	The National Network for Manufacturing Innovation	Carbon fibre composites; lightweight materials; 3D printing technology
US	2019	Nanotechnology Research Plan for 2018–2025	Nanomaterials
Japan	2020	Japan’s Industrial Development Report 2020	Material technology
UK	2020	Sustainable Composites	Full life cycles of composite materials

**Table 3:** Summary of established manufacturing techniques.

Technology	Working Principle	Advantages	Disadvantages	Applications
Surface coating	A film layer is formed on the surface of the substrate	Wide range of optional materials, adaptability to working conditions and good economy	It is difficult to accurately control the film thickness, and subsequent processing is often required	Drug Delivery, corrosion protection, antibacterial activity, pipeline, micro batteries [42]
Additive manufacturing	A “bottom-up” manufacturing method by accumulating materials layer by layer	near net forming, Simple operation	Limited materials, slow manufacturing speed	Biomedical application [43], electronics [44], aerospace applications [45]
High velocity moulding	A technology for consolidating powder by applying pressure of pulse modulated electromagnetic field	Good economy, fast manufacturing and simple operation	Simple structure of parts and low energy utilisation rate	Medical field [46], ceramic [47], packaging material [48]

**Table 4:** Characteristics of various coating technologies.

Surface Coating Technology	Materials	Advantages	Disadvantages	Applications
Plasma spraying [53]	Polymer composite coatings	High bonding strength, simple operation, good adjustment performance	Many interaction parameters, difficult to paint inside holes	Preparation of polymer coatings to improve the corrosion resistance of the substrate
Magnetron sputtering [54]	Polymer metal nanocomposites	Simple equipment, easy to control, strong adhesion, small damage to the substrate, wide range of applicable materials	Difficult and costly preparation of insulator films	Preparation of metal nanofilms on insulating polymer substrates for applications in sensors, reflectors
Electrochemical deposition [55]	Multiwalled carbon nanotubes polymer composites	Simple operation, high flexibility and reliability	Sensitive to the influence of changes in process parameters	Preparation of carbon nanotube-polymer composite films on metal substrates for the protection of metallic materials in ship hulls
Sol–gel technology [56]	Nanostructured polymer composites	Good composition control and film homogenisation ability, low temperature	The drying process is prone to cracking, long moulding cycle and high raw material costs	Preparation of polyimide–silicon dioxide composite coating on epoxy resin substrate to improve corrosion resistance

**Table 5:** Established rapid prototyping technologies.

Technique	State of Starting Materials	Working Principle
FDM [93]	Filament	Extrusion and deposition
SLS [94]	Powder	Laser scanning and heat induced sintering
SLA [95]	Liquid photopolymer	Laser scanning and UV induced curing
3DP [96]	Powder	Drop on demand binder printing

**Table 6:** The different polymer composite manufacturing processes.

Technology	Problem	FE Analysis	Author/Year
Plasma spraying	Simulated crack growth	Discrete element (DEM) model established by C++ program.	Ferguen et al. (2019) [147]
Cold spray	Effect of interfacial bonding on residual stress	Single-particle and multiparticle collision models	Lin et al. (2019) [148]
Cold spray	Effect of process parameters on residual stress	LS-DYNA	Shayegan et al. (2014) [149]
Cold spray	Influence of particle shape on substrate	Smooth Particle Hydrodynamics (SPH)	Yusof et al. (2016) [150]
Plasma spraying	Effect of coating thickness and convective heat transfer coefficient on residual stress of coating	ANSYS	Zhuang et al. (2021) [151]
Modulated pulsed power magnetron sputtering	Effect of sputtering pressure on scratch behaviour	ABAQUS/Explicit	Meng et al. (2019) [152]
Sol–gel	Effect of filler particle size on coating properties	Elastica	Nemeth et al. (2008) [153]
Plasma spraying	Effect of interface morphology on residual stress of coating	ANSYS APDL	Xie et al. (2019) [154]
Cold spray	Effect of particle temperature on bond strength of coating	ABAQUS/Explicit	Song et al. (2021) [155]
Big Area Additive Manufacturing (BAAM)	Thermal evolution	One-dimensional thermal finite difference model	Compton et al. (2017) [156]
FDM (3D)	Thermal gradient and residual stress	ABAQUS	Charoula et al. (2017) [157]
FDM (3D)	Deformations and residual stress distributions of parts during deposition	ANSYS	Ghorbani et al. (2020) [158]
FDM (3D)	Temperature distribution	ANSYS APDL	Ji et al. (2010) [159]
4D printing	Deformation of the designed product	ABAQUS	Yu et al. (2020) [160]
FDM (3D)	Distribution of thermal stress and temperature	ANSYS	Zhou et al. (2017) [161]
3D printing	Predict and compensate deformation	PLM/ABAQUS	Siemens (2019) [162]
5D printing	Conceptual model	--	Reddy PR et al. [123]
Magnetic pulse radial compaction	Influence of process parameters on compactness of compacted parts	ANSYS/Multiphysical and ABAQUS/Explicit	Li et al. (2021) [163]
Magnetic pulse axial compaction	Influence of process parameters on compactness of compacted parts	Theoretical models of pulsed magnetic fields and dynamics of mechanical systems	Olevsky et al. (2013) [164]

**Table 7:** Novel manufacturing technology for polymer composites.

Novel Manufacturing	Technical Difficulties	Advantage	Disadvantage	Application
Surface coating manufacturing [174,175,176,177]	The connection quality of the interface between the spraying material and the base material, and the uniformity, density and mechanical properties of the layer.	High efficiency, simple equipment, easy to achieve batch production.	There are many factors that affect the quality, which are harmful to the environment and operators.	Realise the modification of surface enhancement and modification
Addictive manufacturing [178,179]	Interface bonding strength between layers; Dependence on material size and properties.	No mould is needed to realise moulding of different shapes; low cost and short processing cycle; forming and sintering are completed at the same time.	Low efficiency, low density, low technology maturity, small batch production.	Aerospace parts and human organs with complex structure
Magnetic pulse manufacturing [41,131,132]	Control the discharge energy to reduce the impact on the mould and frame.	High efficiency, cold forming, not easy to carbonise, high density, mass production, products with high mechanical properties and electrical conductivity.	Low energy utilisation rate of equipment; post curing treatment is needed; high requirements for mould quality.	Parts with high density requirements

## Data Availability

Data will be available by contacting the author.
